# Are There Functional Differences in Key Photosynthetic Traits Between Native and Non-Native *Oenothera drummondii* Populations?

**DOI:** 10.3390/plants15213266

**Published:** 2026-10-23

**Authors:** María Zunzunegui, Juan B. Gallego-Fernández, Mari Cruz Díaz-Barradas

**Affiliations:** Departamento de Biología Vegetal y Ecología, Facultad de Biología, Universidad de Sevilla, 41012 Sevilla, Spain; galfer@us.es (J.B.G.-F.); diaz@us.es (M.C.D.-B.)

**Keywords:** A/C_i_ curves, invasive species, light-response curves, *Oenothera drummondii*, photosynthetic capacity, photosynthetic traits, population differentiation

## Abstract

Physiological differentiation between native and introduced populations may facilitate establishment across contrasting environments, but it remains unclear whether this reflects a general enhancement of photosynthetic performance. We investigated light-response traits, photosynthetic biochemical capacity and leaf pigment content in three native and three introduced populations of *Oenothera drummondii* grown under common greenhouse conditions. We measured light-response and A/C_i_ curves with a LI-6400 system and then analyzed them using the Ye and C_3_ biochemical models, respectively. Traits were compared using nested ANOVA, with population nested within origin. Light-response traits differed significantly among populations, but no significant differences between origins were detected. Total chlorophyll content also differed among populations, but no significant origin effect was detected, and it was positively correlated with A_nmax_ and I_max_ across the six populations. Introduced populations had higher net CO_2_ assimilation at external CO_2_ concentrations of 400 and 600 µmol mol^−1^, and higher maximum Rubisco carboxylation and electron-transport capacities. These biochemical capacities did not differ significantly among populations, whereas day respiration showed no significant origin effect. Photosynthetic differentiation therefore involved specific components of photosynthetic performance rather than a uniform enhancement in introduced populations, emphasizing the importance of population-level variation when assessing functional differentiation across geographical ranges.

## 1. Introduction

Many species have been intentionally or unintentionally transported by humans across the world. Some of these species establish, reproduce, and spread in their new environments, often competing with native species and triggering biological invasions. Invasive species are recognized as a major driver of global change [1] and have significant ecological impacts. They can reduce the fitness, abundance, and diversity of native plant and animal species [2], alter ecosystem properties and disturbance regimes [3], disrupt the balance of direct and indirect interactions within the communities [4], and homogenize species pools across habitats in the recipient region [5].

The success of invasive plants is often influenced by environmental variability. Newly established populations may encounter substantial differences in temperature, water availability, and light conditions [6], which can modify their morphology, phenology, and physiology [7]. Additionally, climate change, including elevated temperatures and increased atmospheric CO_2_, can favor invasive species by accelerating growth, modifying ecophysiological responses, or increasing their competitive advantage over native species [8,9,10,11].

Invasive species often differ from co-occurring natives in key functional traits including efficient dispersal mechanisms, higher resource acquisition, superior colonization ability, rapid life cycle and reproduction, faster growth, broad ecophysiological niches, and high environmental tolerance [12,13,14]. In Mediterranean-climate ecosystems, invasive species frequently exhibit traits associated with rapid resource acquisition and high carbon assimilation, although these differences are context-dependent and may vary according to vegetation type [15]. Beyond trait-based differences, invasive success has also been linked to broader physiological niche breadth, with species capable of maintaining performance across a wide range of environmental conditions being more likely to establish and spread in novel environments [16]. However, no universal traits syndrome defines all invasive species [17,18]; instead, different traits may promote invasiveness in different species and communities.

Coastal dunes provide a relevant system in which to examine such differentiation. These ecosystems combine high irradiance, limited water and nutrient availability, salt exposure, sand movement, and recurrent disturbance [19]. These conditions make them particularly susceptible to biological invasions, with invasive species often acting as autogenic ecosystem engineers that modify ecosystem structure through their own biomass and cover [20]. Consequently, coastal dunes are considered among the ecosystems most vulnerable to plant invasions worldwide [21,22].

*Oenothera drummondii* subsp. drummondii Hook. (Onagraceae) is a short-lived perennial herb native to coastal dunes of southeastern North America and the Gulf of Mexico. It has become invasive in Spain, Israel, and China and is naturalized in several other coastal regions worldwide. Its introduced distribution includes climatic conditions that are generally colder and/or drier than those characterizing much of its native range, and the climatic niches occupied in the native and introduced ranges overlap only partially [23]. The species has several characteristics that may facilitate establishment, including a mixed mating system with high self-compatibility and multiple seed-dispersal mechanisms [24,25]. Nevertheless, these characteristics do not explain whether introduced populations have undergone consistent differentiation in photosynthetic function.

Previous studies demonstrate both high ecophysiological performance and marked population dependence in this species. In Mediterranean dunes of southwestern Spain, an introduced population of *O. drummondii* maintained higher CO_2_ assimilation during spring and summer. It showed more effective water regulation than the co-occurring native *Achillea maritima*, consistent with a comparatively rapid resource-acquisition strategy [26]. Greenhouse experiments have also shown variation among populations in responses to drought and salinity. Plants reduced stomatal conductance and CO_2_ assimilation under water deficit but recovered after rewatering, with persistent differences among the populations studied [27]. The species was relatively resistant to salinity, although the native population examined was more tolerant than the introduced population under prolonged exposure and high salt concentrations [28]. Thus, existing evidence does not support a uniform physiological superiority of introduced populations; instead, responses depend on the population, environmental stress, and physiological trait considered.

Population-level differentiation also has a genetic context. Native populations show substantial neutral genetic structuring and marked geographical variation in genetic diversity, with lower diversity in peripheral and southern populations and comparatively higher diversity near the proposed center of origin [29]. These neutral genetic patterns cannot explain photosynthetic variation directly, but they reinforce the importance of treating population identity as a major source of biological variation rather than interpreting every difference solely through the native-introduced contrast.

Although previous studies have documented population-dependent responses to environmental stress, it remains unclear whether native and introduced populations of *O. drummondii* differ consistently in photosynthetic responses to irradiance, biochemical CO_2_-assimilation capacity, or both when grown under common conditions. Here, we grew plants from three native and three introduced populations under common greenhouse conditions and characterized their photosynthetic light- and CO_2_-response curves. We asked: (1) whether light-response traits differ among populations and consistently between native and introduced origins; and (2) whether biochemical parameters derived from A/C_i_ curves show corresponding population- or origin-related differentiation. We predicted substantial differentiation among populations and tested the more specific hypothesis that plants from introduced populations exhibit greater photosynthetic capacity than plants from native populations. By separating responses to irradiance from apparent biochemical capacity for CO_2_ assimilation, this study evaluates whether differentiation associated with geographical origin represents a general enhancement of photosynthetic function or is restricted to particular components of the photosynthetic system.

## 2. Results

### 2.1. Photosynthetic Light Response Curves and Photosynthetic Pigments

Net CO_2_ assimilation increased rapidly at low irradiance and approached saturation at higher PPFD in all six populations (Figure 1). The magnitude of the response differed among populations, despite all plants having been grown under the same greenhouse conditions. At high irradiance (1400 μmol photons m^−2^ s^−1^), net assimilation differed significantly among populations (one-way ANOVA, F_5,23_ = 4.15, *p* = 0.008). DIQ-IM showed the highest mean net assimilation, whereas OJO-NT showed the lowest values (Tukey HSD, *p* = 0.004). Net assimilation rate was positively related to stomatal conductance at PPFDs of 600 and 1400 μmol photons m^−2^ s^−1^ (Figure A1; R^2^ = 0.697 and R^2^ = 0.671, respectively; *p* < 0.001).

Parameters derived from the individual light-response curves differed significantly among populations, except for R_D_ (Table 1). Maximum net assimilation (A_nmax_) at light saturation was highest in DIQ-IM (18.4 ± 1.9 μmol CO_2_ m^−2^ s^−1^) and BOL-NS (17.1 ± 1.7 μmol CO_2_ m^−2^ s^−1^) and lowest in OJO-NT (7.9 ± 0.8 μmol CO_2_ m^−2^ s^−1^). Tukey comparisons indicated that DIQ-IM, ZAR-IA, and BOL-NS differed significantly from OJO-NT, whereas the other populations had intermediate values. Apparent quantum yield at low irradiance (Φ_I0_) was highest in ZAR-IA (0.075 ± 0.005 mol CO_2_ mol^−1^ photons) and lowest in OJO-NT (0.047 ± 0.006 mol CO_2_ mol^−1^ photons); these were the only populations separated by the Tukey test. The light-compensation point (I_comp_) was highest in OJO-NT (18.7 ± 1.1 μmol photons m^−2^ s^−1^) and lowest in MAN-NT (10.6 ± 1.2 μmol photons m^−2^ s^−1^). OJO-NT differed significantly from MAN-NT, while the remaining populations showed intermediate values. The operational light-saturation point (I_max_) was highest in BOL-NS (1348 ± 185 μmol photons m^−2^ s^−1^) and DIQ-IM (1178 ± 59 μmol photons m^−2^ s^−1^), and lowest in OJO-NT (754 ± 43 μmol photons m^−2^ s^−1^) and LEZ-IM (8101 ± 96 μmol photons m^−2^ s^−1^). A similar pattern was observed for I_50_: BOL-NS and DIQ-IM had the highest values (294 ± 73 and 250 ± 13 μmol photons m^−2^ s^−1^, respectively), whereas LEZ-IM had significantly lower values (Table 1).

Despite this population-level variation, none of the light-response parameters differed significantly between native and introduced origins when population was nested within origin (A_nmax_: F_1,4_ = 1.043, *p* = 0.366; Rd: F_1,4_ = 3.99, *p* = 0.114; ΦI_0_: F_1,4_ = 1.83, *p* = 0.245; I_comp_: F_1,4_ = 0.07, a *p* = 0.810; Imax: F_1,4_ = 0.47, *p* = 0.840; I_50_: F_1,4_ = 0.05, *p* = 0.945).

Total chlorophyll content differed significantly among populations (F_5,24_ = 4.09, *p* = 0.008), with BOL-NS showing the highest mean concentration and OJO-NT the lowest (Table 2). Tukey’s post-hoc test indicated significantly higher total chlorophyll content in BOL-NS than in OJO-NT (*p* = 0.012), whereas the remaining pairwise comparisons were not significant. No significant effect of origin was detected on total chlorophyll content (F_1,4_ = 0.19, *p* = 0.687). Across the six population means, total chlorophyll content was positively correlated with A_nmax_ (Pearson’s r = 0.936, *p* = 0.006) and I_max_ (r = 0.979, *p* < 0.001).

### 2.2. Principal Component Analysis and Climatic Relationships of Light Response Traits

The PCA based on the six photosynthetic parameters derived from the light-response curves accounted for 74.8% of the total variance in the first two principal components (PC1 = 43.8% and PC2 = 31%; Figure 2). PC1 was mainly associated with A_nmax_ and I_max_, which showed negative loadings in the same direction. PC2 primarily reflected variation in Φ_I0_, and R_d_, which showed positive loadings, whereas I_50_ was negatively associated with this component (Table A1). Native and introduced individuals overlapped in multivariate space, indicating no clear differentiation in light-response traits by origin (Figure 2), consistent with the nested ANOVA’s lack of a significant origin effect.

Regarding climatic variables, exploratory analyses showed that several photosynthetic traits derived from the light-response curves were negatively correlated with annual precipitation at the population sites. These relationships were statistically significant for A_nmax_ and Φ_Io_ (both at *p* < 0.05).

### 2.3. A/C_i_ Response Curves

Net CO_2_ assimilation increased with intercellular CO_2_ concentration in all six populations, although substantial variation was observed both among and within populations (Figure 3). Introduced individuals showed higher net CO_2_ assimilation than native individuals at the two external CO_2_ concentrations selected for comparison. At C_a_ = 400 μmol CO_2_ mol^−1^, introduced individuals had significantly higher net photosynthetic rates than native individuals (20.3 ± 1.78 vs. 15.3 ± 1.40 μmol CO_2_ m^−2^ s^−1^, respectively; F_1,4_ = 8.42, *p* = 0.044, values are means ± SE). This difference was also observed at C_a_ = 600 μmol CO_2_ mol^−1^, where introduced individuals showed higher net photosynthetic rates than native individuals (27.6 ± 2.47 vs. 19.8 ± 1.77 μmol CO_2_ m^−2^ s^−1^, respectively; F_1,4_ = 13.13, *p* = 0.022, values are means ± SE). No significant differences among the six populations in net CO_2_ assimilation were detected at either external CO_2_ concentration (*p* > 0.05). Net CO_2_ assimilation was significantly associated with stomatal conductance at both external CO_2_ concentrations, showing a linear relationship (with R^2^ = 0.69 at C_a_ = 400 µmol CO_2_ mol^−1^ and R^2^ = 0.67 at C_a_ = 600 µmol CO_2_ mol^−1^; *p* < 0.01).

Parameters derived from the individual A/C_i_ curves are summarized in Table 3. Population means ranged from 72.5 ± 23.2 to 143.2 ± 32.7 μmol m^−2^ s^−1^ for the maximum Rubisco carboxylation rate (V_Cmax_), from 92.2 ± 24.0 to 170.0 ± 76.1 μmol electrons m^−2^ s^−1^ for maximum electron transport rate (J_max_), from 6.0 ± 1.5 to 10.9 ± 4.7 μmol m^−2^ s^−1^ for triose phosphate utilization (TPU), and from 1.5 ± 0.4 to 2.9 ± 1.6 μmol CO_2_ m^−2^ s^−1^ for respiration in the light (R_d_).

None of the parameters differed significantly among the six sampled populations (Table 3). However, nested ANOVAs, with population nested within origin, showed that introduced populations had significantly higher V_Cmax_ (*F*_1,4_ = 9.76, *p* = 0.035), J_max_ (*F*_1,4_ = 14.69, *p* = 0.018) than native populations, whereas R_d_ did not differ significantly between origins (*F*_1,4_ = 3.25, *p* = 0.145). Estimated TPU was also significantly higher in introduced populations (*F*_1,4_ = 13.64, *p* = 0.020). However, TPU was estimated from the highest C_i_ point without establishing a clearly defined TPU-limited region; this result should therefore be regarded as exploratory. Overall, introduced populations exhibited higher values for specific components of biochemical photosynthetic capacity than native populations (Figure 4).

The relationship between V_Cmax_ and J_max_ showed a similar pattern in native and introduced populations, indicating a shared coordination of photosynthetic capacity regardless of origin (Figure 5).

### 2.4. Principal Component Analysis and Climatic Relationships of A/C_i_-Derived Traits

The PCA based on the four photosynthetic parameters derived from the A/C_i_ curves accounted for 99.0% of the total variance in the first two principal components (PC1 = 91.8% and PC2 = 7.2%; Figure 6). PC1 represented a general gradient of photosynthetic biochemical capacity, with V_Cmax_, J_max_, TPU, and R_d_ showing positive loadings in the same direction. PC2 primarily reflected variation in R_d_, which showed a strong negative loading on this component, whereas V_Cmax_, J_max_, and TPU showed weaker positive loadings (Table A2). Native and introduced individuals overlapped in multivariate space, although introduced individuals tended towards higher PC1 scores, consistent with their higher V_Cmax_, J_max,_ and TPU values.

Relationships between A/C_i_-derived parameters and climatic variables were explored using the six population means (n = 6). V_Cmax_ was negatively correlated with mean annual precipitation (*p* = 0.003), whereas the corresponding relationship for J_max_ did not reach statistical significance (*p* = 0.070). R_d_ was negatively correlated with mean annual temperature (*p* = 0.048). Given the small number of populations, the number of climatic relationships examined, and the partial confounding between climate and biogeographical origin, these associations were considered exploratory and were not interpreted as evidence of climatic adaptation.

## 3. Discussion

Our results revealed substantial population-level variation in the photosynthetic physiology of *Oenothera drummondii* under common greenhouse conditions. However, this variation was not consistently associated with native or introduced origin. Light-response traits varied markedly among populations but showed no overall differentiation between origins, either individually or in multivariate trait space. In contrast, introduced populations showed higher CO_2_ assimilation at the external CO_2_ concentrations examined and differences in several parameters derived from A/C_i_ curves. Thus, our hypothesis of enhanced photosynthetic performance in introduced populations was only partially supported, with origin-related differentiation being more apparent in CO_2_ assimilation capacity than in the response to irradiance.

Across all populations, we observed a positive linear relationship between A_n_ and gs in both light- and A/C_i_-response curves, indicating a close coupling between stomatal conductance and photosynthetic carbon assimilation under the well-watered conditions of the experiment. This strong coordination suggests that stomatal regulation was an important component of photosynthetic performance. However, the higher CO_2_ assimilation rates observed in introduced individuals, together with their higher V_Cmax_ and J_max_, suggest that differences in biochemical photosynthetic capacity also contributed to origin-related variation.

### 3.1. Population Differentiation in Light-Response Traits

Light-response curves presented the classical saturation pattern characteristic of C_3_ species and were well described by the Ye model [30,31]. The marked variation in light-response traits was primarily population-specific. DIQ-IM, ZAR-IA, and BOL-NS showed comparatively high maximum net assimilation rates, whereas OJO-NT exhibited the lowest A_nmax_ and Φ_I0_. Light requirements for photosynthetic saturation also varied markedly among populations, with the highest I_max_ values observed in BOL-NS and DIQ-IM and the lowest in OJO-NT and LEZ-IM. Similarly, I_50_ was highest in BOL-NS and DIQ-IM and lowest in LEZ-IM. Importantly, these differences did not follow a consistent native–introduced pattern: both native and introduced populations occurred among those showing relatively high and low values. The positive correlations between total chlorophyll content and both A_max_ and I_max_ suggest that variation in pigment content may contribute to population-level differences in photosynthetic responses to irradiance. Associations between leaf traits, including chlorophyll concentration, and light-response parameters have also been reported across herbaceous and woody angiosperms [32]. However, the associations observed here were based on six population means and should therefore be interpreted cautiously.

Accordingly, none of the light-response parameters differed significantly between native and introduced populations when population-level variation was accounted for. Consistent with these results, the PCA showed extensive overlap between native and introduced individuals, with no clear separation according to biogeographical origin. However, origin comparisons were based on only three populations per group, with four denominator degrees of freedom, limiting statistical power to detect differences. Therefore, the non-significant origin effects should not be interpreted as evidence of equivalence between native and introduced populations. Broader population sampling would be needed to assess whether this pattern holds across the native and introduced ranges.

Some of the population-level variation was associated with climatic conditions at the sites of origin. In particular, A_nmax_ and Φ_I0_ were negatively related to annual precipitation, suggesting that populations originating from drier environments tended to exhibit greater photosynthetic capacity and light-use efficiency under the common greenhouse conditions. However, the combination of traits observed in individual populations was complex. For example, OJO-NT, originating from the wettest native site (>2000 mm annual precipitation, compared with approximately 500 mm at Mediterranean sites), combined low A_nmax_, Φ_I0_, and I_max_ with the highest I_comp_. Although this population did not show a low light compensation point, it exhibited the lowest light saturation point, indicating that photosynthetic saturation was reached at comparatively lower irradiance [33,34]. Thus, its overall combination of light-response traits precludes a simple interpretation as a low-light-adapted photosynthetic strategy. Moreover, because climatic relationships were based on only six population localities and climate was partially confounded with geographical origin, these associations should be regarded as exploratory rather than as evidence of climatic adaptation.

### 3.2. CO_2_ Assimilation and Biochemical Capacity

Regarding A/C_i_ curves, A_n_ increased approximately linearly until an external CO_2_ concentration of 400 μmol mol^−1^, corresponding to C_i_ values of approximately 250 to 280 μmol mol^−1^. Above this range, the slope decreased, although photosynthetic assimilation remained high at elevated CO_2_ concentrations. This pattern is consistent with the expected transition from predominantly Rubisco-limited assimilation at lower C_i_ towards increasing limitation by RuBP regeneration as C_i_ increases. At high C_i_, some introduced populations maintained assimilation rates above 25 µmol m^−2^ s^−1^, comparable to values reported for the invasive *Acacia* species [35], highlighting the high photosynthetic capacity reached by *O. drummondii* under elevated CO_2_ availability.

Plants from introduced populations showed higher mean net assimilation at C_a_ = 400 and 600 μmol mol^−1^. This greater CO_2_ assimilation capacity contrasts with the lack of a statistically origin effect on light-response parameters, suggesting that physiological differentiation between native and introduced populations was more evident in their response to CO_2_ availability than in their response to irradiance.

Consistent with this pattern, nested ANOVAs accounting for population-level variation showed significantly higher apparent V_Cmax_ and J_max_ in introduced than in native populations. These differences indicate greater apparent Rubisco carboxylation and electron-transport capacities in introduced populations and may contribute to their higher assimilation rates under increased CO_2_ availability. TPU estimates were also significantly higher in introduced populations, but their interpretation is constrained by the measurement limitations discussed below. In contrast, R_d_ did not differ significantly between origins, providing no evidence for an origin-related shift in respiratory carbon costs. Thus, origin-related differentiation appears to involve specific components of biochemical CO_2_-assimilation capacity rather than a generalized enhancement of photosynthetic performance.

Higher biochemical photosynthetic capacity has been reported in several invasive plant species, with high V_Cmax_ and J_max_ associated with rapid resource acquisition and high photosynthetic performance [36,37,38,39,40,41,42]. In this context, the higher V_Cmax_ and J_max_ observed in introduced *O. drummondii* populations are consistent with physiological patterns reported for some other invasive species. However, in the present study this differentiation was restricted to specific components of CO_2_ assimilation capacity and did not extend to the suite of light-response traits.

These biochemical parameters should nevertheless be interpreted with some caution. Because mesophyll conductance was not measured, C_i_ rather than chloroplastic CO_2_ concentration (C_c_) was used to derive the A/C_i_ parameters; therefore, the estimated V_Cmax_ and J_max_ should be interpreted as C_i_-based estimates. As the A/C_i_ curves extended to an external CO_2_ concentration of 600 μmol mol^−1^, TPU was estimated from the highest Ci point available. This CO_2_ range may not have been sufficient for all individuals to reach a clearly defined TPU-limited region. Although estimated TPU differed significantly between origins, this difference should be interpreted cautiously and does not, by itself, demonstrate greater carbon export or sink capacity in introduced populations.

Despite these methodological considerations, the relatively high V_Cmax_ and J_max_ values estimated for *O. drummondii* also indicate a substantial biochemical capacity for carbon assimilation. Values reported across a broad range of plant species average approximately 64 μmol m^−2^ s^−1^ for V_Cmax_ and 134 μmol m^−2^ s^−1^ for J_max_ [43], while lower V_Cmax_ values have been reported for several Mediterranean woody species, including *Pistacia lentiscus* [44] and Mediterranean ash and oak species [45]. The comparatively high values observed in *O. drummondii*, particularly in introduced populations, are therefore consistent with a high biochemical capacity for photosynthetic carbon assimilation.

Higher apparent V_Cmax_ and J_max_ could increase carbon gain under favorable conditions, potentially providing more resources for growth and reproduction and thereby contributing to establishment and spread. Griffin-Nolan et al. [46] found that invasive plants in open habitats increased their investment in Rubisco in their introduced range relative to their native range, enhancing carboxylation rates. Our results suggest a potential physiological contribution to invasion success, although nitrogen allocation, growth, reproductive performance, and competitive interactions were not assessed in this study.

### 3.3. Relationship with Previous Ecophysiological Evidence

The present study formed part of a broader research program on *O. drummondii* that examined its impacts on coastal dune communities [22], geographical distribution [23], floral traits [7,25], seed dispersal [24], genetic variation [29], and ecophysiological responses [26,27,28]. Here, we extend this work by comparing photosynthetic light responses and apparent biochemical CO_2_-assimilation capacity across native and introduced populations under common greenhouse conditions.

Previous studies show that *O. drummondii* can maintain high physiological performance in Mediterranean coastal dunes, but they also reveal substantial variation among populations and experimental conditions. In a field comparison in south-western Spain, one introduced population of *O. drummondii* maintained higher assimilation during spring and summer and more effective water regulation than the co-occurring native species *Achillea maritima* [26]. That interspecific comparison supports the capacity of *O. drummondii* to perform effectively in Mediterranean dunes, but it does not demonstrate that introduced populations generally outperform native populations of the same species.

Population dependence is also evident in experimental responses to drought and salinity. Plants from native and introduced populations reduced gas exchange under water deficit and recovered effectively after rewatering, although the magnitude of their responses differed among populations [27]. Under salinity, one native population was more resistant to prolonged exposure and high salt concentrations than the introduced population examined [28]. Together with the present results, which revealed strong population-level variation in light-response traits but origin-related differentiation in specific components of CO_2_-assimilation capacity, these studies argue against a simple hierarchy in which introduced populations consistently show superior physiological function. Instead, relative performance appears to depend on population identity, environmental conditions, and the physiological component considered.

### 3.4. Genetic Context and Interpretation of Common-Environment Differences

The persistence of population differences under common greenhouse conditions indicates that they were not solely determined by the immediate growth environment. However, because plants were obtained directly from field-collected seeds, genetic differentiation cannot be separated from maternal or other transgenerational environmental effects. Therefore, the observed differences should not be interpreted as evidence of local adaptation, which would require reciprocal environmental experiments and demonstration of associated fitness advantages.

Previous evidence also supports substantial population differentiation in *O. drummondii*. Differences in floral traits have been reported between native and introduced populations along a latitudinal gradient [22], while neutral genetic differentiation has been reported among native populations of *O. drummondii* [29]. More recent population genetic analyses, including the same six populations examined here, revealed substantial genetic structuring both between native and non-native ranges and among populations within each range (Hernández-Espinosa et al., submitted). This provides an independent context for the strong population-level variation observed here, although neutral genetic differentiation cannot be causally linked to photosynthetic traits. Moreover, origin was partially confounded with geography and climate, and the observed physiological differences may therefore reflect a combination of historical, geographical, and environmental factors rather than the introduction process alone, particularly given the limited number of populations sampled from each origin.

## 4. Materials and Methods

### 4.1. Study Species

*Oenothera drummondii* subsp. *drummondii* Hook. (Onagraceae), commonly known as beach evening primrose, has erect to procumbent, densely pubescent stems and a strong taproot. It occurs mainly on back beaches and dune ridges under warm-temperate, subtropical, and tropical climates [23,47,48]. Its natural habitat comprises exposed sandy dune systems subjected to wind erosion, sand deposition, large temperature fluctuations, salt spray, and low soil moisture [48].

The species produces large yellow actinomorphic flowers and has a mixed mating system involving both self- and cross-pollination, with a high degree of self-compatibility [25]. Its dehiscent capsules contain numerous small seeds with high germination capacity [7]. Seeds can disperse through barochory, thalassochory and endozoochory; marine currents may contribute to regional spread, whereas hares and rabbits can act as local dispersal vectors in invaded dune systems [24].

### 4.2. Seed Collection and Plant Cultivation

Six populations of *Oenothera drummondii* were included in the study: three from the native range and three from the introduced range (Table 4). This balanced design allowed comparisons between origins while incorporating population-level variation within each origin. The native populations were distributed along the Gulf of Mexico, from Bolivar in the north through La Mancha to Ojoshal in the south, encompassing contrasting geographical and climatic conditions. The introduced populations comprised two populations from Mediterranean-climate regions, located in south-western Spain (Dique) and Israel (Lezion), and one population from an Atlantic-climate region in northern Spain (Zarautz). These introduced sites were selected because they supported large, established populations and previous ecological and ecophysiological information was available [27]. The collection sites encompassed substantial variation in mean annual temperature and precipitation and represented different Köppen climate types [23]. Populations were coded using site abbreviations followed by origin and climate (Table 4): native tropical (NT), native subtropical (NS), introduced Mediterranean (IM), and introduced Atlantic (IA).

Mature fruits were collected in situ from at least 30 randomly selected individuals per population in July–September 2016. Seeds were pooled within each population and stored in paper bags under laboratory conditions at room temperature until germination.

In December 2016, approximately five seeds were sown directly in 2.5-L pots containing a 70:30 (*v*/*v*) mixture of sand and perlite and were germinated under greenhouse conditions at the University of Seville, Spain. Following emergence, the first seedlings to become established and develop their first leaves were retained, and the remaining seedlings were removed, leaving one plant per pot. Greenhouse air temperature ranged from 15 to 20 °C and relative humidity from 40 to 60%. Natural daylight was supplemented with artificial lighting to provide a 14-h photoperiod. Plants were fertilized in April, May, and September 2017 with a commercial liquid fertilizer (Flower; N:P 6:4:6). On each occasion, every pot received 250 mL of a solution containing 5 mL fertilizer L^−1^ water, equivalent to 1.25 mL of fertilizer per pot. In June 2017, ten plants were selected from each population, providing an initial pool of 60 plants for subsequent physiological measurements. This pool included reserve individuals to allow replacement in the event of mortality or deterioration in plant condition, to measure five plants per population for each curve type.

### 4.3. Measurements of Light- and CO_2_-Response Curves

Light-response and A/C_i_ curves were measured using a LI-6400 portable photosynthesis system (Li-Cor Inc., Lincoln, NE, USA) on young, fully expanded leaves. For each curve type, five plants per population were randomly selected from the initial pool of ten individuals. Selection was conducted separately for the two curve types, resulting in partially overlapping sets of plants, with two or three individuals per population used for both light-response and A/C_i_ measurements. The final light-response dataset comprised 29 plants: five per population except BOL, for which only four curves were available. The A/C_i_ dataset comprised 30 plants, with five individuals per population. One plant from MAN-NT was measured twice for A/C_i_ curves, and the results of the two measurements were averaged to obtain a single estimate per individual. For light response curves, leaves were acclimated at a CO_2_ concentration of 400 μmol mol^−1^ and maintained at a chamber temperature of 27 °C and a relative humidity > 60%. After 15 min of acclimation, photosynthetic photon flux density (PPFD) was decreased stepwise from 2000 to 0 μmol photons m^−2^ s^−1^ (2000, 1800, 1600, 1400, 1200, 1000, 800, 600, 500, 400, 300, 200, 100, 50, 0). Net CO_2_ assimilation rates (A_n_), transpiration rates (E), and stomatal conductance (g_s_) were recorded after stabilization at each light level (coefficient of variation < 2%). Leaf area was determined from scanned images using Midebmp 4.2 software (R. Ordiales, CSIC, Almería, Spain, 2000), and all measurements were expressed on a leaf area basis. Light response measurements were conducted over a 15-day period in June–July 2017.

A/C_i_ curves were measured over a 15-day period in September 2017 under similar conditions. Cuvette conditions were maintained at a PPFD of 1600 µmol m^−2^ s^−1^, relative humidity > 60%, and leaf temperature of 27 °C. Ambient CO_2_ concentration (C_a_) in the cuvette was controlled using a CO_2_ mixer and adjusted sequentially to 400, 300, 200, 100, 50, 400, 400, 500, 600 μmol CO_2_ mol^−1^. The first measurement after returning to 400 μmol mol^−1^ was used for reacclimation and the second for subsequent analyses. Gas exchange parameters (A_n_, E, and g_s_) were recorded once steady-state conditions were reached (coefficient of variation < 2%). Measurements were expressed on an area basis. Five plants per population were used for A/C_i_ curves.

### 4.4. Photosynthetic Pigment Determination

Young, fully expanded, healthy leaves were collected from five plants per population selected for light-response measurements. A subsample was weighed within 1 h of collection to determine fresh mass, then dried at 70 °C for 48 h and reweighed to determine dry mass. The resulting dry-to-fresh mass ratio was used to express pigment concentrations on a dry mass basis. The remaining leaf material was used to quantify photosynthetic pigments following Lichtenthaler [49]. Chlorophyll a (Chl a), chlorophyll b (Chl b), and total carotenoids (Car) were extracted with 100% acetone and quantified spectrophotometrically. Pigment concentrations were expressed on a dry mass basis (mg g^−1^ dry mass).

### 4.5. Analysis of Light Response Curves

Photosynthetic light-response curves were analyzed using the Ye model [30,31,50,51], fitted individually to each plant using non-linear optimization in R. This model describes the increase in net photosynthetic rate at low irradiance and allows for a decline in photosynthesis at high irradiance. The model was expressed as Equation (1):

(1) $$A_{n}=\alpha \times \frac{1-\beta \times I}{1+\gamma \times I}-R_{D}$$

where A_n_ is the net CO_2_ assimilation rate (μmol CO_2_ m^−2^ s^−1^), α is the initial quantum yield (Φ_I0_, mol CO_2_ mol^−1^ photons); I is the photosynthetic photon flux density (PPFD, μmol photons m^−2^ s^−1^); β and γ are coefficients describing the response of photosynthesis to increasing irradiance; and R_D_ is the dark respiration rate (μmol CO_2_ m^–2^ s^−1^). From the fitted curves, additional physiological parameters were derived, including maximum net photosynthetic rate (A_nmax_, μmol CO_2_ m^−2^ s^−1^), the light compensation point (I_comp_, the irradiance at which A_n_ = 0, μmol photons m^−2^ s^−1^), and I_50_ (the irradiance at which A_n_ reaches 50% of its maximum). A_nmax_ was determined as the maximum of the fitted function or, for asymptotic curves, as its limiting value.

The light saturation point (I_max_) was estimated following Lobo et al. [51] and defined as the irradiance beyond which no further significant increase in A_n_ occurs, using a fixed increment of 50 μmol photons m^−2^ s^−1^. Instantaneous quantum yield at the compensation point (Φ_Icomp_) was calculated as the first derivative of the fitted curve evaluated at I_comp_. In addition, mean quantum yields were estimated as the slopes of linear regressions of A_n_ over defined irradiance intervals: Φ_Io-Icomp_, representing the mean efficiency between darkness and I_comp_, and Φ_Icomp-I200_, representing the mean efficiency between I_comp_ and 200 μmol photons m^−2^ s^−1^.

### 4.6. Analysis of CO_2_-Response Curves (A/C_i_)

Photosynthetic CO_2_ response curves (A/C_i_) were analyzed to estimate key photosynthetic parameters and indices of photosynthetic capacity using the biochemical model of C_3_ photosynthesis developed by Farquhar et al. [52] and further refined by Sharkey [53]. Parameter estimation was performed in R-4.4.2 using a simplified linear fitting approach described by Sharkey [54,55], in which different limitation phases of the A/C_i_ curve were analyzed separately. According to this model, net CO_2_ assimilation (A_n_) may be limited by: (i) the carboxylation capacity of Rubisco (at C_i_ < 200 µmol mol^−1^), (ii) the rate of ribulose-1,5-bisphosphate (RuBP) regeneration via electron transport, or (iii) the rate of triose phosphate utilization (TPU).

For each individual curve, the maximum Rubisco carboxylation rate (V_Cmax_) and day respiration (Rd) were estimated by fitting a linear regression to the Rubisco-limited portion of the A/Ci curve (Ci < 200 µmol mol^−1^.), using the Rubisco kinetic constants (K_c_, K_o_, Γ*) adjusted to a leaf temperature of 27 °C following the temperature response functions described by Bernacchi et al. [56]. The rate of electron transport (J) was estimated from measurements at C_i_ ≥ 250 µmol mol^−1^ using the RuBP-regeneration-limited equation of the Farquhar model, and J_max_ was estimated as the mean J across the available measurements within this high-Ci range. Triose phosphate utilization (TPU) was estimated from the measurement at the highest C_i_ for each individual curve following the simplified approach described by Sharkey et al. [54]. Because the A/C_i_ curves extended only to an external CO_2_ concentration of 600 µmol mol^−1^, a clearly defined TPU-limited region may not have been reached in all individuals; TPU values should therefore be regarded as estimates based on the highest available Ci measurements.

When photosynthesis is Rubisco-limited, the response of A_n_ to CO_2_ concentration can be described by the following Equation (2):

(2) $$A_{n}=\;V_{cmx}\frac{C_{i}-\;\Gamma^{*}}{C_{i}+\;K_{c\;}\left(1+\frac{O}{K_{o}}\right)}-R_{d}$$

where V_Cmax_ is the maximum carboxylation rate of Rubisco, *C_i_* is the intercellular CO_2_ concentration, Γ* is the CO_2_ compensation point in the absence of mitochondrial respiration, *K_c_* and *K_o_* are the Michaelis–Menten constants of Rubisco for CO_2_ and O_2_, respectively, *O* is the O_2_ partial pressure, and *R_d_* is the day respiration rate. Because mesophyll conductance was not estimated, intercellular CO_2_ concentration (*C_i_*) was used as a proxy for chloroplast CO_2_ concentration (C_c_).

When photosynthesis is limited by RuBP regeneration, A_n_ is described by the Farquhar-von Caemmerer-Berry model (3) as follows [52,57,58]:

(3) $$A_{n}=\;J\frac{C_{i}-\;\Gamma^{*}}{4C_{i}+\;{8\Gamma }^{*}}-R_{d}$$

where *J* is the rate of electron transport, assuming that four electrons are required per carboxylation or oxygenation event.

### 4.7. Data Analysis

Photosynthetic parameters obtained from the light-response and A/C_i_ curves, together with leaf pigment contents, were compared between origins (native vs. introduced) using nested analyses of variance (ANOVAs), with population nested within origin. The effect of origin was tested against variation among populations within origin, thereby accounting for the hierarchical sampling design and treating populations as the appropriate level of replication for origin-level comparisons. Differences among the six populations in photosynthetic traits derived from the light-response and A/C_i_ curves were assessed using one-way ANOVAs. When significant differences were detected, Tukey’s HSD post-hoc test was used for pairwise comparisons. Model assumptions were assessed using the Shapiro–Wilk test applied to model residuals. Bivariate Pearson correlation analyses were used to examine the relationship between net photosynthetic rate and stomatal conductance, as well as associations of photosynthetic parameters with total chlorophyll content and climatic variables at the population sites. Correlations involving total chlorophyll content and s climatic variables were examined using population means (n = 6) and were considered exploratory because of the small number of populations and the partial confounding between climate and biogeographical origin. When significant relationships were detected, linear regression analyses were performed. Principal component analyses (PCA) were used as a descriptive complement to the univariate ANOVAs to summarize covariation among photosynthetic traits and to visualize patterns of individual variation associated with population and biogeographical origin. For the light-response PCA, six of the 12 derived parameters (A_nmax_, Φ_I0_, R_d_, I_comp_, I_50_, and I_max_) were retained. The remaining five parameters were excluded to reduce redundancy because each was strongly correlated with at least one of the retained variables (r > 0.86). The A/C_i_-response PCA was based on four derived photosynthetic parameters (V_Cmax_, J_max_, TPU, and R_d_). Variables were centered and scaled to unit variance prior to PCA. Individuals were represented in the space defined by the first two principal components, with population identity and origin indicated graphically. Variable loadings were used to interpret the contribution of individual photosynthetic traits to each principal component.

## 5. Conclusions

The contrasting patterns observed in light- and CO_2_-response traits reveal that photosynthetic differentiation in *O. drummondii* depends on the physiological component considered. Responses to irradiance were predominantly structured at the population level, whereas differentiation between native and introduced populations was more evident in specific components of biochemical CO_2_-assimilation capacity. This dissociation argues against a uniform enhancement of photosynthetic performance following introduction and instead highlights the importance of population-level variation. Nevertheless, the greater CO_2_-assimilation capacity observed in introduced populations may represent a functional advantage, and may contribute to the assimilation of biomass and growing capacity, although its contribution to invasion success remains to be established.

The persistence of substantial population-level variation under common greenhouse conditions indicates that these differences cannot be attributed solely to the immediate environments experienced by the plants. Given the documented impacts of *O. drummondii* on native communities [22], these findings emphasize the value of incorporating intraspecific variation when investigating invasion processes across geographical ranges. Future research combining physiological measurements with genomic analysis, broader population sampling and multigenerational experiments would help distinguish inherited differentiation from maternal and other transgenerational effects. Experiments across contrasting environmental conditions would allow explicit evaluation of phenotypic plasticity, while field experiments measuring growth, reproduction, and fitness would help establish whether the observed physiological differences contribute to establishment and invasion success.

## Figures and Tables

**Figure 1:**
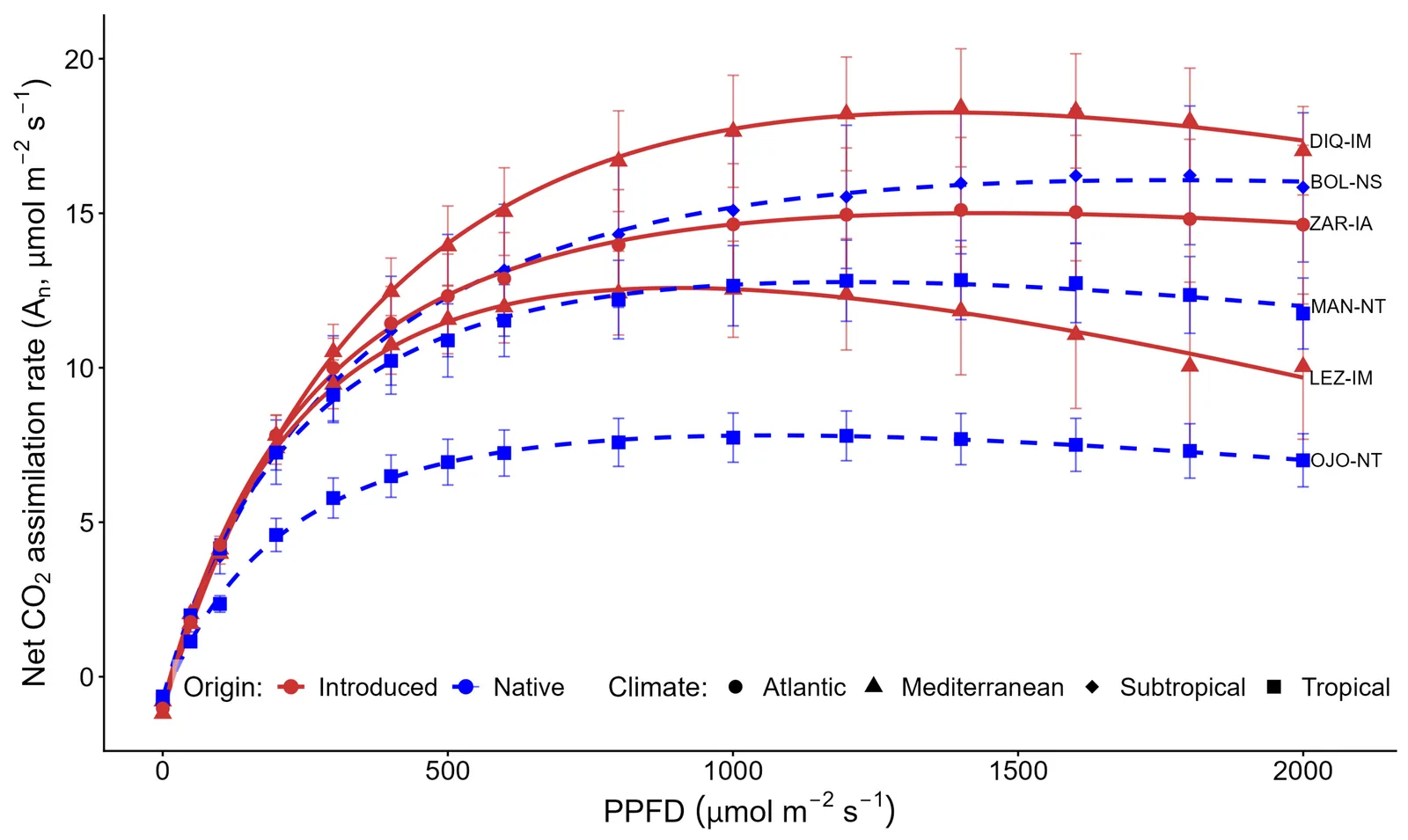
Light-response curves for the six *Oenothera drummondii* populations studied. Points represent means ± SE of net photosynthetic assimilation rates (A_n_ ± SE) at different photosynthetic photon flux densities (PPFD) for each population. Curves were fitted to pooled individual measurements within each population using the Ye model [30,31], as described in Materials and Methods. Model parameters used for the statistical analyses were estimated separately for each individual plant. Blue dashed lines represent native populations, and red solid lines represent introduced populations. Population codes indicate the study site, origin (N = native and I = introduced), and climatic region (M = Mediterranean, A = Atlantic, S = Subtropical, and T = Tropical).

**Figure 2:**
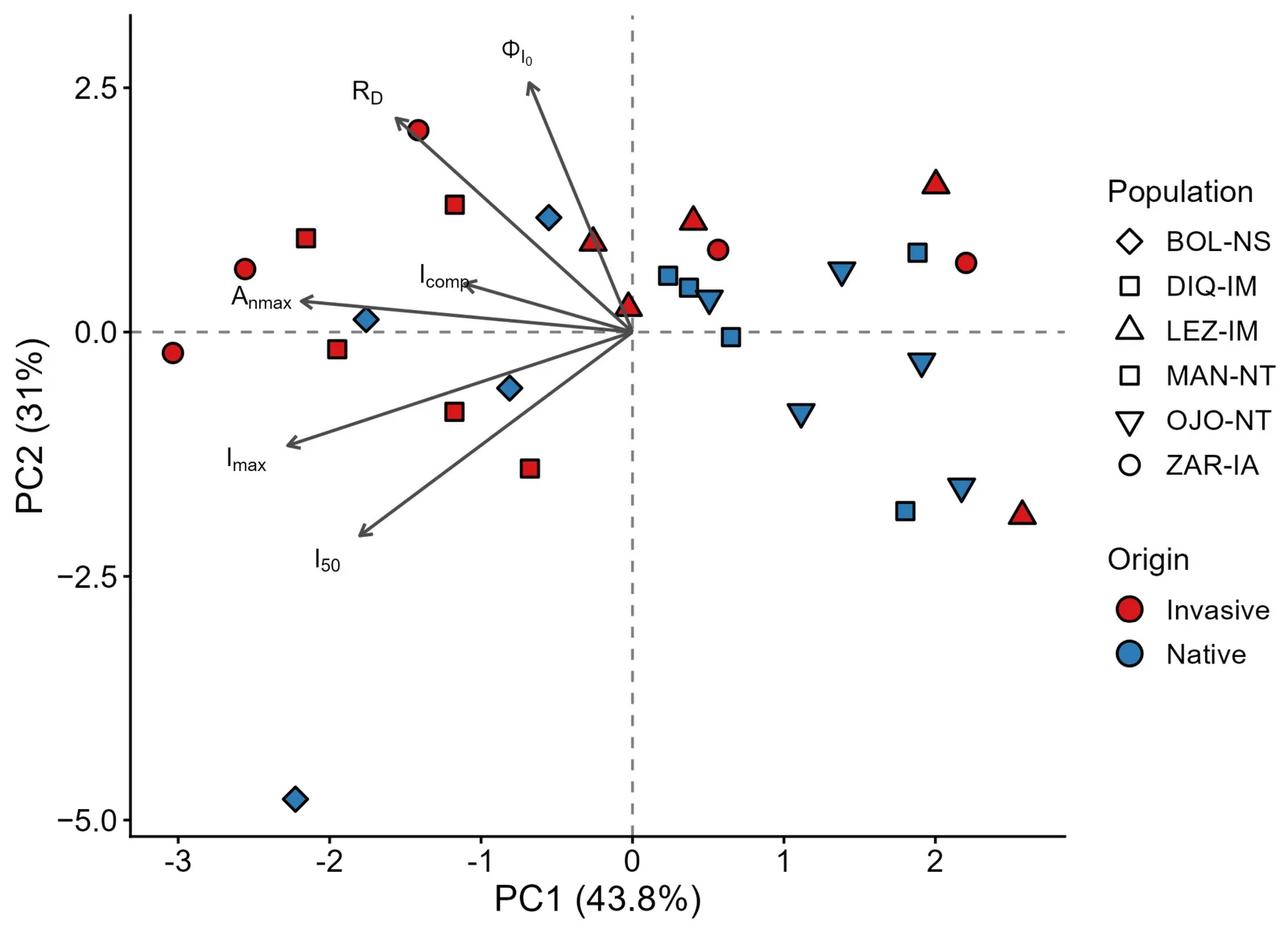
Principal component analysis (PCA) of photosynthetic parameters derived from the light-response curves. Points represent individual plants, colored by origin (native in blue and introduced in red) and shaped by population. The PCA was based on seven photosynthetic parameters, with variables centered and scaled before analysis: maximum net assimilation rate at light saturation (A_nmax_), apparent quantum yield at zero irradiance (Φ_I0_), dark respiration (R_D_), the irradiance at which net assimilation is zero or light compensation point (I_comp_), irradiance at which photosynthesis reaches 50% of maximum net photosynthesis (I_50_), and the light-saturation point (I_max_). Arrows indicate the direction and relative contribution of each variable to the first two principal components.

**Figure 3:**
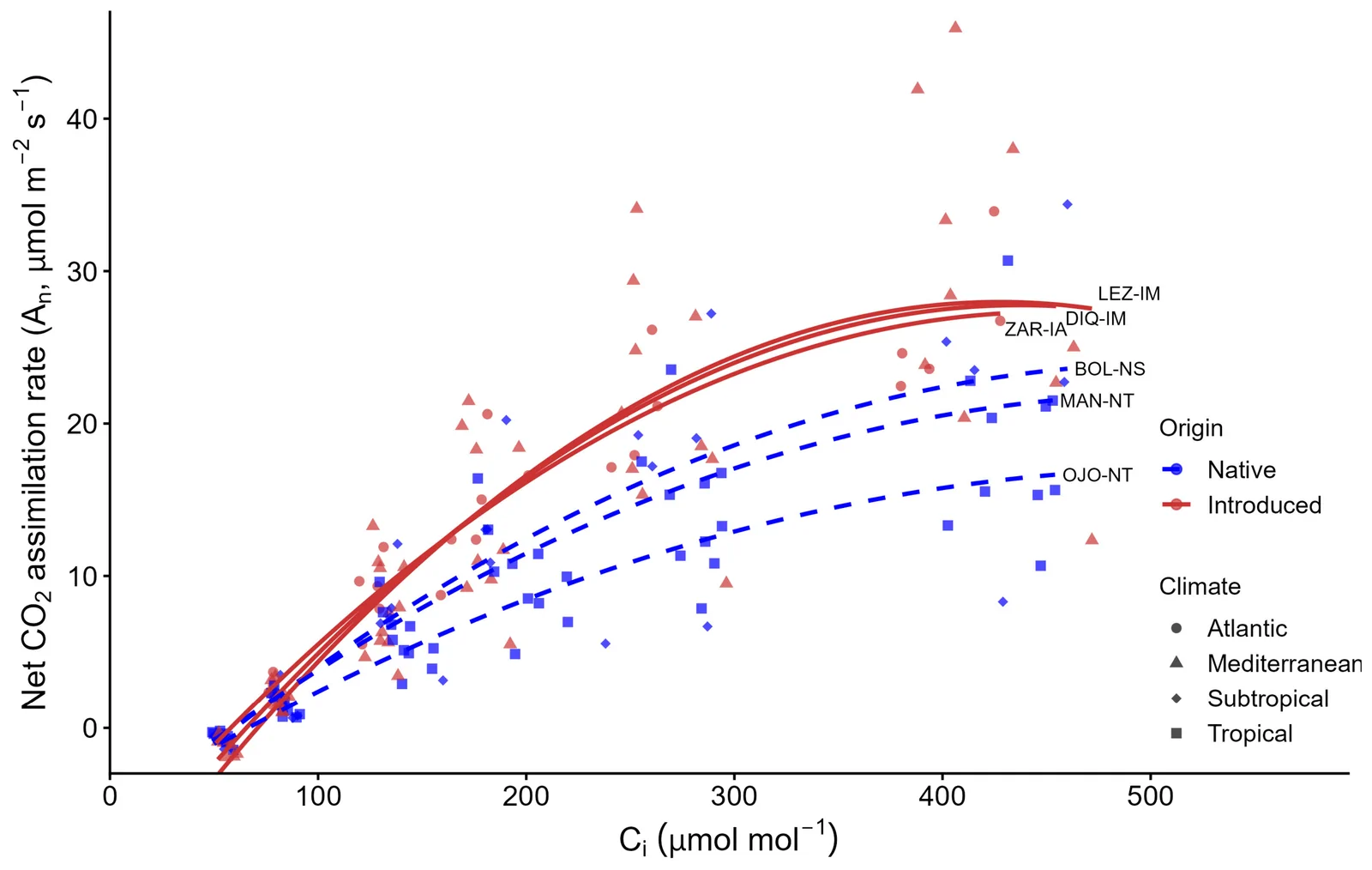
A/C_i_-response curves for the six *O. drummondii* study populations. Points represent individual leaf-level measurements of net photosynthetic rates across a range of intercellular CO_2_ concentrations (C_i_) for each population (n = 5 individuals per population). Curves were fitted at the population level using a second-degree polynomial model, as described in the Materials and Methods. Blue dashed lines represent native populations, whereas red solid lines represent invasive populations. Population codes indicate the study site, origin (N = native and I = invasive), and climatic region (M = Mediterranean, A = Atlantic, S = Subtropical, and T = Tropical).

**Figure 4:**
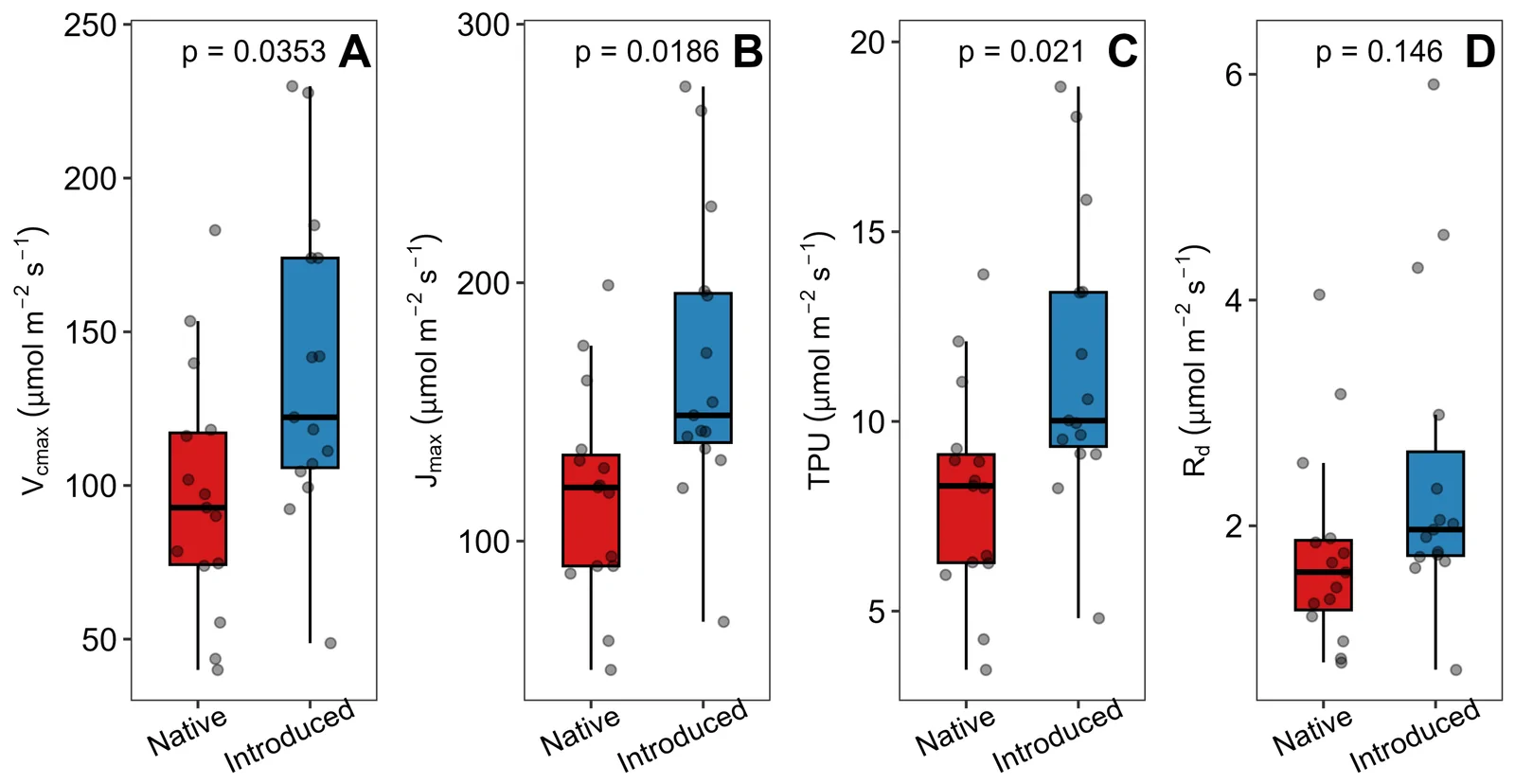
Photosynthetic parameters derived from A/C_i_ curves in native and invasive *Oenothera* populations. (**A**) Maximum Rubisco carboxylation rate (V_Cmax_), (**B**) maximum electron transport rate (J_max_), (**C**) estimated triose phosphate use (TPU), and (**D**) day respiration rate (R_d_). Boxplots indicate the median and interquartile range, and points represent individual plants (n = 15 invasive and n = 15 native individuals). Colours denote origin (native in blue and invasive in red). *p*-values in each panel indicate the results of linear mixed-effects models testing the effect of origin, with population included as a random effect.

**Figure 5:**
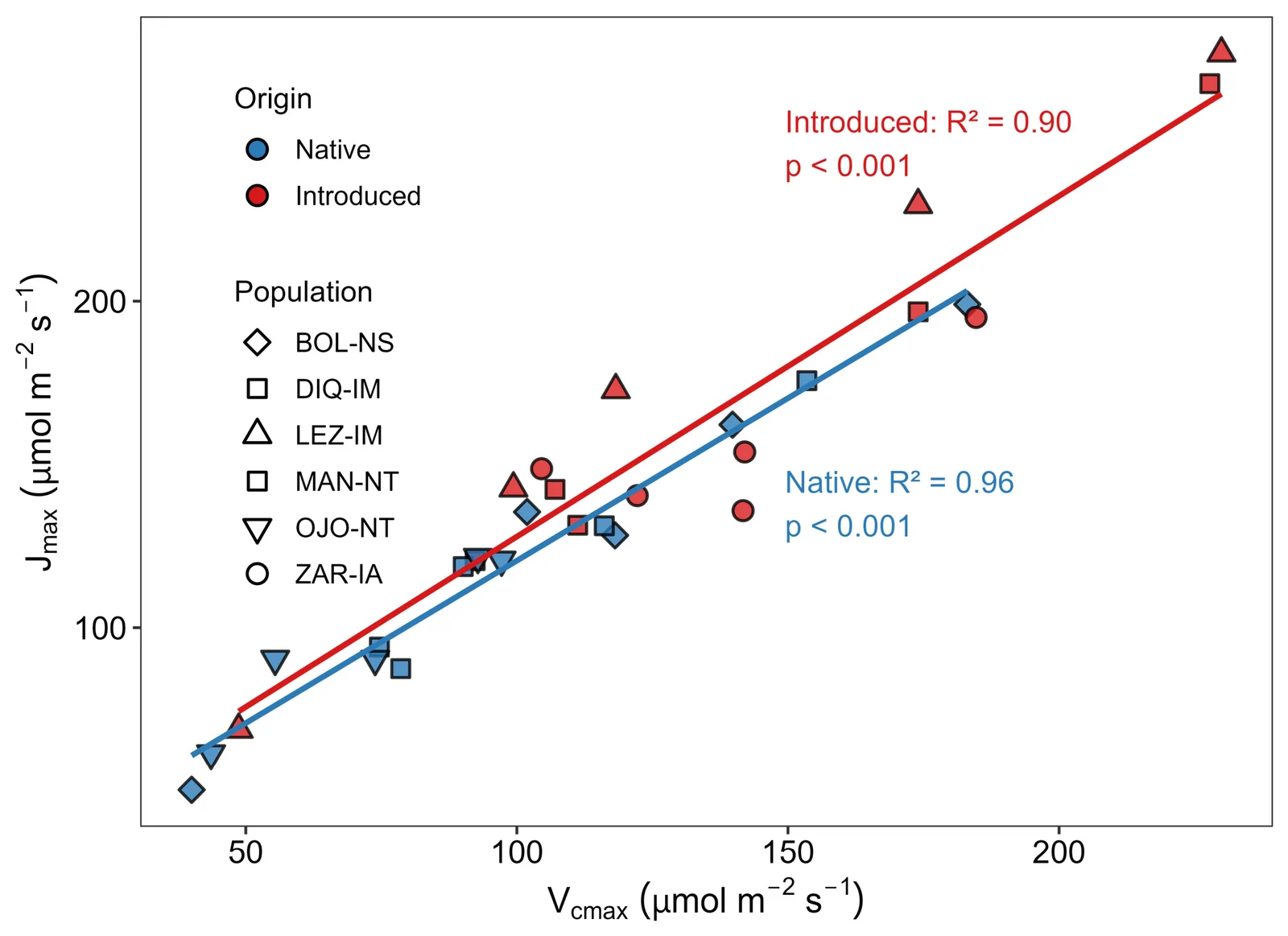
Relationship between maximum Rubisco carboxylation rate (V_Cmax_) and maximum electron transport rate (J_max_) across *Oenothera* populations. Points represent individual plants (n = 15 invasive and n = 16 native individuals); colors indicate origin, and shapes represent specific populations. Solid lines indicate linear regressions for each origin. R^2^ and *p*-values are shown for each group.

**Figure 6:**
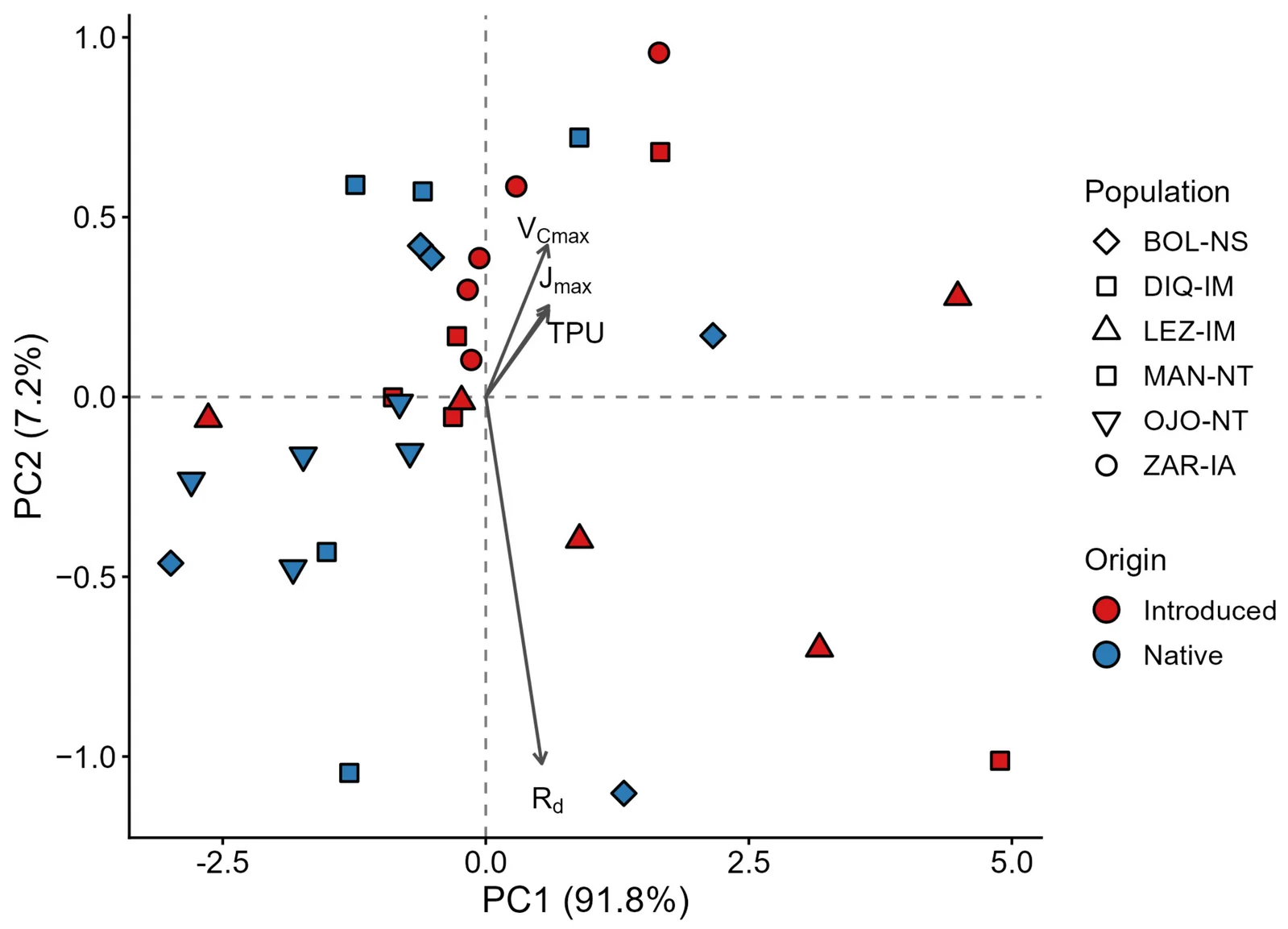
Principal component analysis (PCA) of photosynthetic parameters derived from the A/C_i_ curves. Points represent individual plants (n = 5 per population), colored by origin (native in blue and invasive in red) and shaped by population. The PCA was based on four photosynthetic parameters: maximum Rubisco carboxylation rate (V_Cmax_), maximum electron transport rate (J_max_), estimated triose phosphate utilization (TPU), and respiration in the light (R_d_).

**Table 1:** Mean (±SE) photosynthetic traits derived from light-response curves for each population. Parameters include maximum net assimilation rate at light saturation (A_nmax_, μmol CO_2_ m^–2^ s^−1^), apparent quantum yield at low light (Φ_I0_, mol CO_2_ mol photons ^−1^), dark respiration (R_D_, μmol CO_2_ m^–2^ s^−1^), the irradiance at which net assimilation is zero or light compensation point (I_comp_, μmol photon m^–2^ s^−1^), irradiance at which photosynthesis reaches 50% of maximum net photosynthesis (I_50_, μmol photon m^–2^ s^−1^), and the light-saturation point (I_max_, μmol photon m^–2^ s^−1^). F and *p* values correspond to one-way ANOVA testing the effect of population on each photosynthetic parameter. Significant *p* values are shown in bold. Different letters indicate significant differences among populations according to Tukey’s HSD test (*p* < 0.05).

Population	A_nmax_	R_D_	Φ_I0_	I_comp_	I_max_	I_50_
DIQ-IM	18.4 ± 1.9 a	1.11 ± 0.14	0.062 ± 0.004	18.5 ± 1.7 ab	1178 ± 59 ab	250 ± 13 a
LEZ-IM	13.0 ± 1.5 ab	0.82 ± 0.15	0.069 ± 0.007	11.9 ± 1.8 bc	810 ± 96 ab	165 ± 16 b
ZAR-IA	15.6 ± 2.3 a	1.16 ± 0.12	0.075 ± 0.005	16.4 ± 1.7 abc	1140 ± 211 ab	201 ± 31 ab
BOL-NS	17.1 ± 1.7 a	0.88 ± 0.18	0.061 ± 0.010	15.0 ± 1.7 abc	1348 ± 185 a	294 ± 73 a
MAN-NT	12.8 ± 1.3 ab	0.70 ± 0.10	0.068 ± 0.007	10.6 ± 1.2 c	907 ± 45 ab	170 ± 13 ab
OJO-NT	7.9 ± 0.8 b	0.81 ± 0.09	0.047 ± 0.006	18.7 ± 1.1 a	754 ± 43 b	166 ± 9 b
F_5,23_	5.24	2.01	2.2	4.76	3.02	3.57
*p*	**0.0023**	0.1158	0.0896	**0.0039**	**0.0305**	**0.0156**

**Table 2:** Leaf photosynthetic pigment concentrations and chlorophyll a/b ratios in the six populations of *Oenothera drummondii*. Values are means ± SE (n = 5 plants per population). Pigment concentrations are expressed in mg g^−1^ dry mass. Letters indicate significantly different according to Tukey’s HSD test (*p* < 0.05). F and *p* values correspond to one-way ANOVAs testing differences among populations. Significant *p* values are shown in bold.

Population	Chl a	Chl b	Total chl	Carotenoids
DIQ-IM	2.79 ± 0.22 ab	0.96 ± 0.08 ab	3.76 ± 0.29 ab	0.96 ± 0.07 a
LEZ-IM	2.04 ± 0.23 ab	0.69 ± 0.07 ab	2.74 ± 0.30 ab	0.71 ± 0.07 ab
ZAR-IA	2.77 ± 0.24 ab	0.93 ± 0.09 ab	3.70 ± 0.32 ab	0.92 ± 0.06 ab
BOL-NS	3.06 ± 0.39 a	1.00 ± 0.14 a	4.06 ± 0.52 a	1.01 ± 0.12 a
MAN-NT	2.30 ± 0.22 ab	0.76 ± 0.08 ab	3.05 ± 0.30 ab	0.80 ± 0.06 ab
OJO-NT	1.70 ± 0.16 b	0.58 ± 0.06 b	2.28 ± 0.22 b	0.58 ± 0.06 b
F_5,24_	4.25	3.6	4.09	4.58
*p*	**0.0066**	**0.0144**	**0.008**	**0.0045**

**Table 3:** Photosynthetic parameters derived from A/C_i_ curves for each population: maximum Rubisco carboxylation rate (V_Cmax_, μmol CO_2_ m^−2^ s^−1^), maximum electron transport rate (J_max_, μmol electrons m^−2^ s^−1^), triose phosphate utilization (TPU, μmol m^−2^ s^−1^), and day respiration rate (R_d_, μmol CO_2_ m^−2^ s^−1^). Values are means ± SE (n = 5 plants per population).

Population	V_Cmax_	J_max_	TPU	R_d_
DIQ-IM	142.5 ± 25.5	171.5 ± 27.2	11.7 ± 1.8	2.7 ± 0.8
LEZ-IM	134.1 ± 31.3	178.0 ± 35.7	12.2 ± 2.4	2.9 ± 0.7
ZAR-IA	139.0 ± 13.4	154.8 ± 10.5	10.5 ± 0.8	1.8 ± 0.1
BOL-NS	116.6 ± 23.5	135.0 ± 24.6	9.3 ± 1.7	2.2 ± 0.6
MAN-NT	102.6 ± 14.6	121.4 ± 15.7	8.4 ± 1.1	1.6 ± 0.3
OJO-NT	72.6 ± 10.4	96.9 ± 11.2	6.7 ± 0.8	1.5 ± 0.2
F_5,24_	1.61	1.87	1.83	1.28
*p*	0.195	0.137	0.145	0.306

**Table 4:** Geographic location and climatic data of native and non-native populations of *Oenothera drummondii*. Climatic data were obtained from the WorldClim website. Köppen climatic classification is also included [7]. Native tropical (NT), native subtropical (NS), introduced Mediterranean (IM), and introduced Atlantic (IA).

Population	Acronym	Country	Origin	Latitude	Longitude	Temp °C	Ppt mm	Köppen
Ojoshal	OJO-NT	Mexico	Native	18°15′ N	93°59′ W	25.6	2059	Am Tropical
La Mancha	MAN-NT	Mexico	Native	19°37′ N	96°22′ W	25.7	1124	Aw Tropical
Bolivar	BOL-NS	USA	Native	29°30′ N	94°30′ W	20.8	1100	Cfa Subtropical
Dique	DIQ-IM	Spain	Invasive	37°09′ N	06°54′ W	17.8	473	Csa Mediterranean
Zarautz	ZAR-IA	Spain	Invasive	43°17′ N	02°09′ W	14.6	1259	Cfb Atlantic
Rishon-Lezion	LEZ-IM	Israel	Invasive	31°59′ N	34°43′ E	19.9	553	Csa Mediterranean

**Figure A1:**
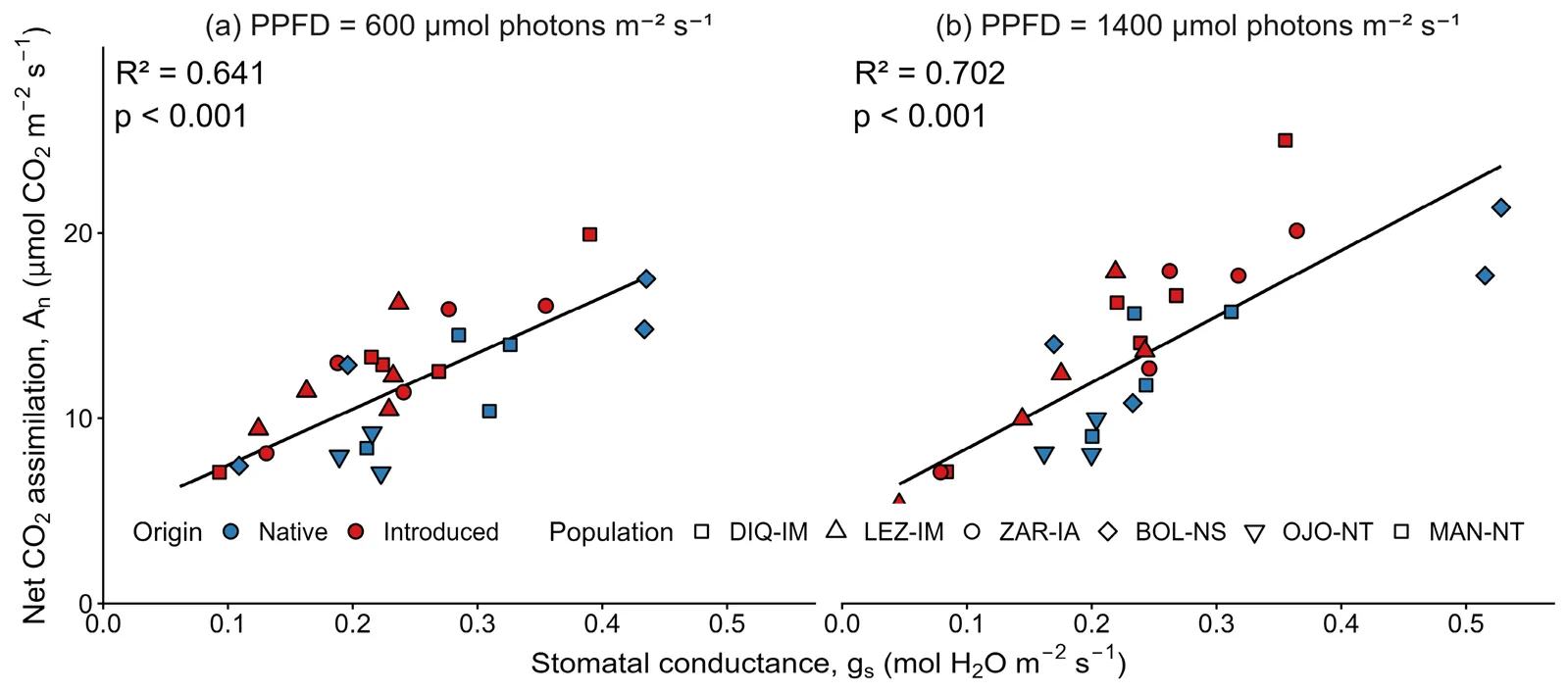
Relationships between net CO_2_ assimilation (An) and stomatal conductance (g_s_) at PPFDs of (**a**) 600 and (**b**) 1400 µmol photons m^−2^ s^−1^ in *Oenothera drummondii*. Points represent individual plants (n = 29), colored by origin (native in blue and introduced in red) and shaped by population. Black lines represent linear regressions fitted across all populations within each panel.

## Data Availability

The datasets supporting this study’s findings, including gas-exchange data and derived photosynthetic parameters, are available from the corresponding author upon reasonable request.

## Abbreviations

A_n_	Net CO_2_ assimilation
A_gmax_	Maximum gross photosynthetic rate
A_n max_	Maximum net assimilation rate at light saturation
C_a_	Ambient CO_2_ concentration
C_i_	Intercellular CO_2_ concentration
g_s_	Stomatal conductance
E	Transpiration rates
Φ_Icomp_	Instantaneous quantum yield at the compensation point
Φ_I0_	Apparent quantum yield at low light
Φ_Io-Icomp_	Mean quantum yields efficiency between darkness and I_comp_
Φ_Icomp-I200_	Mean quantum yields efficiency between I_comp_ and 200 μmol photons m^−2^ s^−1^.
R_D_	Dark respiration for the light-response non rectangular hyperbolic model
I_comp_	The irradiance at which net assimilation is zero or light compensation point
I_50_	Irradiance at which photosynthesis reaches 50% of maximum net photosynthesis
I_max_	Light-saturation point
θ	Dimensionless convexity parameter of the non-rectangular hyperbola
V_Cmax_	Maximum Rubisco carboxylation rate
J_max_	Maximum electron transport rate
TPU	Triose phosphate utilization
R_d_	Day respiration rate for the Farquhar biochemical model of C_3_ photosynthesis
OJO-NT	Ojoshal native area with tropical climate
MAN-NT	La Mancha native area with tropical climate
BOL-NS	Bolivar native area with subtropical climate
DIQ-IM	Dique introduced area with Mediterranean climate
ZAR-IA	Zarautz introduced area with Atlantic climate
LEZ-IM	Rishon-Lezion introduced area with Mediterranean climate

## Appendix A

**Table A1:** Loadings of photosynthetic parameters on the first two principal components (PC1 and PC2) of the PCA based on the light response-curve parameters. Maximum net assimilation rate at light saturation (A_nmax_), apparent quantum yield at low light (Φ_I0_,), dark respiration (R_D_,), the irradiance at which net assimilation is zero or the light compensation point (I_comp_), irradiance at which photosynthesis reaches 50% of maximum net photosynthesis (I_50_), and the light-saturation point (I_max_).

Variable	PC1	PC2
A_nmax_	−0.525	0.076
Φ_Io_	−0.164	0.613
R_D_	−0.375	0.526
I_comp_	−0.266	0.119
I_50_	−0.432	−0.501
I_max_	−0.547	−0.279
Variance (%)	43.9	31.0

**Table A2:** Loadings of photosynthetic parameters on the first two principal components (PC1 and PC2) of the PCA based on A/C_i_ response-curve parameters. Maximum Rubisco carboxylation rate (V_Cmax_) maximum electron transport rate (J_max_), triose phosphate utilization (TPU), and day respiration rate (R_d_).

Parameter	PC1	PC2
V_Cmax_	0.505	0.364
J_max_	0.517	0.209
TPU	0.516	0.219
R_d_	0.46	−0.881
Variance (%)	91.7	7.2
